# Genetic and environmental aetiologies of the transition from nonsuicidal self-injury to suicide attempt: a longitudinal twin study

**DOI:** 10.1038/s41380-025-03165-z

**Published:** 2025-08-22

**Authors:** Anna Ohlis, Lin Li, Ralf Kuja-Halkola, Sebastian Lundström, Brian M. D’Onofrio, Clara Hellner, Paul Lichtenstein, Martin Cederlöf, Zheng Chang, Johan Bjureberg

**Affiliations:** 1 Department of Clinical Neuroscience, Centre for Psychiatry Research, Karolinska Institutet, & Stockholm Health Care Services, Region Stockholm, Stockholm, Sweden; 2 Department of Global Public Health, Karolinska Institutet, Stockholm, Sweden; 3 Department of Medical Epidemiology and Biostatistics, Karolinska Institutet, Stockholm, Sweden; 4 Department of Neuroscience and Physiology, Sahlgrenska Academy, Gothenburg, Sweden; 5 Department of Psychological and Brain Sciences, Indiana University, Bloomington, IN USA; 6 Department of Clinical Science, Intervention and Technology, Karolinska Institutet, Stockholm, Sweden; 7 School of Medical Sciences, Faculty of Medicine and Health, Örebro University, Örebro, Sweden

**Keywords:** ADHD, Genetics

## Abstract

Nonsuicidal self-injury (NSSI) often temporally precedes suicide attempts (SA), and SA predicts suicide. The genetic and environmental aetiologies of the transition from NSSI to SA have not been studied. This study aims to investigate whether NSSI reported at age 18 influences the incidence of SA between ages 18 and 24, and to what extent these transitions from NSSI to SA are influenced by shared genetic and environmental factors. Twins born in Sweden were enrolled in this longitudinal population-based twin cohort study. Self-reports of NSSI and SA were collected at ages 18 and 24. The majority of individuals in the analytical sample (N = 3 934) were female (64.9%) and dizygotic twins (65.0%). We found that NSSI reported at age 18 was associated with an increased risk of SA between ages 18 and 24 (Odds Ratio 5.4, 95% CI 3.3–8.7), after adjusting for sex and childhood psychopathology. There was a strong genetic correlation between NSSI reported at age 18 and incidence of SA between ages 18 and 24 (rA=0.8, 95% CI 0.3–1.0). At age 18, the proportion of variance in NSSI explained by genetic factors was 53%, and the remaining variance was explained by non-shared environmental factors (47%). At age 24, genetic factors explained 30% of the variance in SA between ages 18 and 24, largely explained by shared genetic factors (66.6%) with NSSI reported at age 18. We found evidence that NSSI reported at age 18 had a strong genetic correlation with incidence of SA between ages 18 and 24.

## Introduction

Non-suicidal self-injury (NSSI) and suicide attempts (SA) are prevalent in youth and predict subsequent suicide [1–3]. According to the WHO, suicide is the fourth leading cause of death globally among youth 15 – 19 years of age [4].

Longitudinal studies have shown that NSSI often temporally precedes SA [3, 5–11], which in turn predicts suicide [1, 3]. However, not all individuals who engage in NSSI attempt suicide. Based on a range of studies [5, 12–14], Griep and MacKinnon [7] stated in their review that between 6% and 37% of those who engage in NSSI also attempt suicide.

Genetic studies (family, twin- and genome-wide association studies) have shown an extensive genetic correlation between different psychiatric disorders [15–17], and also between NSSI and SA [18], and NSSI and suicidal ideation [19]. Assuming that environmental risk factors do not differ systematically for monozygotic (MZ) versus dizygotic (DZ) twins, twin studies make it possible to distinguish to what extent a shared trait between individuals – or individual differences in a trait – is due to genetic versus environmental factors [17]. Moreover, a longitudinal twin design can show to what extent a transition from one trait to another is genetically driven. A better understanding of the transition from NSSI in adolescence to SA in early adulthood, in particular why some individuals are more prone to proceed from NSSI to SA, may offer new opportunities in preventing suicide [20].

The current study presents the results from the first longitudinal twin study investigating the genetic and environmental contribution of the transition from NSSI to SA, conducted with the following aims:

1. To study the extent to which NSSI reported at age 18 is associated with incidence of SA between ages 18 and 24.
2. To investigate to what extent the transitions from NSSI reported at age 18 to SA between ages 18 and 24 are influenced by shared genetic and environmental factors.

## Methods

### Participants

The Child and Adolescent Twin Study in Sweden (CATSS) is an ongoing longitudinal study including all twins born in Sweden since July 1, 1992 [21]. CATSS uses both parent and youth report questionnaires to collect information on various psychological constructs and medical conditions (overall response rate: 80%) [21]. In our study, the sample comprised 4 748 youth who participated in CATSS both at ages 18 and 24. Due to missing values on items related to NSSI or SA, n = 581 (12%) were excluded from the analysis. A total of 233 (4.9%) individuals reported SA at age 18 were further excluded. Thus, we include 3 934 individuals in the population-level regression analysis, and 1 719 complete twin pairs (3 438 individuals) in the twin-specific analyses. Informed consent was obtained from all subjects. The study was approved by the Regional Research Ethical Committee in Stockholm (DNR 2016/2135-31) and conducted in accordance with the Swedish Ethical Review Act (2003:460) and the principles of the Declaration of Helsinki. All methods were performed in accordance with the relevant guidelines and regulations.

### Measures

#### Exposure

The exposure was defined as ever having engaged in NSSI at age 18. At ages 18 and 24, youth completed the Lifetime History of Aggression (LHA) questionnaire, which contained an assessment of NSSI and SA. The LHA included the following two questions on self-harm: (Q1): ‘How many times in life so far, would you say that you have deliberately attempted to injure yourself physically when you were angry or despondent?’ and (Q2): ‘How many times in life so far, would you say that you have deliberately attempted to kill yourself when you were angry or despondent?’ The original response scale included ‘Never’ (0), ‘One Event’ (1), ‘2–3 Events (2), ‘4–9 Events’ (3), ‘10+ Events’ (4), and ‘More times than I can count’ (5). We dichotomized each item into absent (0) or ever present (1). We defined NSSI reported at age 18 as having NSSI (Q1) without SA (Q2). Individuals with SA reported at age 18 were excluded.

#### Outcomes

The main outcome identified at age 24 was SA, with or without NSSI (CATSS-24: Q2 = 1).

#### Covariates

We adjusted for potential confounding due to psychopathology broadly presenting in childhood. At age 9/12, parents completed the Autism–Tics, AD/HD, and other Comorbidities (A-TAC) Inventory concerning each child. The A-TAC is a validated and comprehensive screening interview designed to assess autism spectrum disorders, attention deficit/hyperactivity disorder (ADHD), tic disorders, developmental coordination disorder, learning disorders, and other childhood mental disorders [22, 23]. Parents could indicate their response to each item as ‘Yes’ (1), ‘Yes, to a certain extent’ (0.5), or ‘No’ (0). We calculated the total scores by summing the items for the following domains as suggested by previous studies [24]: concentration/attention (ranging from 0 to 9), impulsivity/activity (0 to 10), opposition (0 to 5), conduct (0 to 5), eating (0 to 2), and reality/psychosis (0 to 1).

### Statistical analysis

#### Regression analyses

We used logistic regression models to estimate the association between NSSI reported at age 18 and SA reported at age 24. We accounted for the non-independence of observations in families by using a cluster-robust sandwich estimator for standard errors. Sex and A-TAC scores were adjusted for in the adjusted models.

#### Concordance rates and tetrachoric correlations

The twin method relies on comparing genetically identical MZ twin pairs, who share 100% of their segregating alleles, with DZ twin pairs, who share an average 50% of their segregating alleles. Therefore, only twin pairs with known zygosity were included in the analyses. Before variance decomposition, we first estimated the tetrachoric correlations for NSSI reported at age 18 and SA reported at age 24. Higher concordance rates and correlations of liability in MZ than in DZ twins indicate a genetic contribution to the manifestation of disease. Intra-class correlation (ICC; refers to correlation of a trait, i.e. separately for NSSI reported at age 18 and for SA reported at age 24, between twins in pairs) described similarity in one trait between twins in a pair and could imply genetic liability for the disorder if estimated higher in MZ twins than in DZ twins. Cross-twin cross-trait correlation (CTCT, correlation of one trait in one twin and the other trait in his/her co-twin) describes the overlap between the two traits. These correlations described the relationships between traits within MZ and DZ twins, respectively. Shared environmental influences were implied if the DZ correlation was greater than half the MZ correlation. If CTCT MZ > DZ it implies genetic correlation between NSSI reported at age 18 and SA reported at age 24.

#### Structural equation modelling

Structural equation modelling in the statistical software R version 4.2.2, with the OpenMx package [25] was used to conduct two univariate and one bivariate quantitative genetic model for NSSI reported at age 18 and SA reported at age 24, and their combinations. Quantitative genetic modeling decomposes variance of each disorder and covariance between two disorders into additive genetic effects (A), shared environmental effects (C), and unique environmental effects (E, including measurement error). We compared the goodness of fit of the ACE- (i.e., model including A, C, and E components) and AE-models, and calculated the genetic and environmental variance components from the best-fitting model. We used a liability-threshold approach, assuming that the observed binary variable came from an underlying continuous liability of the trait [26]. A threshold was assumed, and a 1 was assigned if an individual had a liability greater than the threshold, and 0 otherwise. The underlying liabilities were assumed to have normal distributions, and the (tetrachoric) correlations between these underlying normal distributions were estimated [27].

In the bivariate model, we assess the relative contribution of A, C, and E to each trait, as well as the genetic (rA), shared environmental (rC), and nonshared environmental (rE) correlations, The explained variance in the outcome can be partitioned into A, C and E unique to the outcome and A, C and E shared with the predictors.

## Results

We found that among 18-year-old twins, 21.0% had ever engaged in NSSI. By the age of 24, 2.7% of twins had attempted suicide (excluding those who had ever attempted suicide at age 18, n = 233 [4.9%]). As shown in Table 1, the majority of individuals were female (64.9%) and DZ twins (65.4%). The means of A-TAC scores ranged from 0.01 for reality to 0.7 for attention problems.

**Table 1:** Descriptive of included individuals (N = 3 934, N-Pairs = 1 719).

Variable		N (%)
NSSI at age 18	Yes	826 (21.0)
	No	3 108 (79.0)
SA at age 24	No SA	3 360 (97.3)
	SA, with or without NSSI	92 (2.7)
Sex	Male	1 521 (38.7)
	Female	2 413 (64.9)
Zygosity	MZ	1 360 (34.7)
	DZ	2 559 (65.0)
	Unknown	15 (0.4)
A-TAC scores		Mean ± SD
Attention		0.7 ± 1.4
Impulsive		0.7 ± 1.4
Opposition		0.4 ± 0. 8
Conduct		0.2 ± 0.6
Reality		0.01 ± 0.1
Feeding		0.1 ± 0.2

*NSSI* nonsuicidal self-injury, *SA* suicide attempt, *MZ* monozygotic twins, *DZ* dizygotic twins. *A-TAC* Autism-Tics, ADHD, and other Comorbidities.

NSSI reported at age 18 was associated with increased risk of SA reported at age 24 (OR 5.4, 95% CI 3.3–8.7), after adjusting for sex and A-TAC scores (see Table 2).

**Table 2:** The associations between NSSI at age 18 and risk of SA at age 24.

	Number of events	Odds Ratio (95%CI)	
	SA at age 24	Crude	Adjusted^a^
NSSI at age 18	54	6.33 (4.14–9.68)	5.37 (3.32–8.69)
No NSSI at age 18	38	Reference	Reference

Analysis was done by logistic models.

*NSSI* nonsuicidal self-injury, *SA* suicide attempt. *CI* Confidence interval.

^a^Adjusted for sex, Autism-Tics, ADHD, and other Comorbidities (A-TAC) items.

As shown in Table 3, ICC for NSSI reported at age 18 and SA reported at age 24 were higher in MZ twins than in DZ twins, suggesting genetic effects on each trait given that MZ twins share more genetic variance than DZ twins. The CTCTs between NSSI reported at age 18 and SA reported at age 24 were also higher in MZ twins than in DZ twins, suggesting genetic effects on the observed associations. Similar results were found for both males and females (Supplementary Table 1).

**Table 3:** Tetrachoric correlations for NSSI at age 18 and risk of SA at age 24.

	NSSI at age 18		SA at age 24		Tetrachoric correlations		
	Both affected/both unaffected^a^	Discordant pairs^b^	Both affected/both unaffected	Discordant pairs	ICC NSSI at age 18	ICC SA at age 24	CTCT
All pairs	90/1194	435	4/1 594	121	–	–	–
MZ twins	51/469	142	2/620	40	0.58 (0.46–0.70)	0.35 (−0.06–0.76)	0.33 (0.15–0.51)
DZ twins	39/723	293	2/973	80	0.14 (0.007–0.27)	0.09 (−0.23–0.41)	0.13 (−0.02–0.28)

*NSSI* nonsuicidal self-injury, *SA* suicide attempt, *ICC* intraclass correlation, *CTCT* cross-twin cross-trait, *MZ* monozygotic, *DZ* dizygotic.

^a^Number of pairs with both twins affected versus (/) number of pairs with both twins unaffected by the trait.

^b^Number of pairs where one twin was affected with the trait and the other was unaffected.

**Fig. 1:**
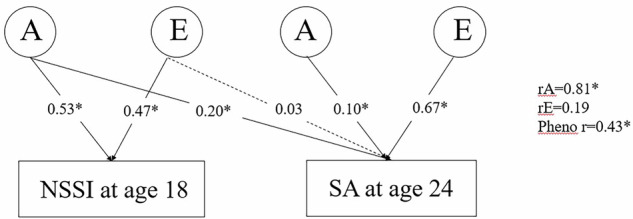
Additive genetic (A) and non-shared Environmental (E) Cholesky decomposition of the relationship between the nonsuicidal self-injury (NSSI) at age 18 and suicide attempt (SA) at age 24. Numbers on arrows are explained variance. Genetic (rA), environmental (rE), and phenotypic (pheno r) correlations are shown to the right of the path model. *P < 0.05.

Heritability was estimated to be 53% in NSSI reported at age 18 and, 30% in SA reported at age 24, the remaining variance was mainly explained by non-shared environmental factors, shared environmental factors did not contribute (Table 4 and Supplementary Table 2). In bivariate analysis, we found high genetic correlations between NSSI reported at age 18 and SA reported at age 24 (rA = 0.81; Fig. 1). Specifically, 75% of the correlation between NSSI reported at age 18 and SA reported at age 24 was explained by shared genetic factors, indicating a significant genetic overlap between NSSI reported at age 18 and SA reported at age 24.

**Table 4:** Estimates of genetic and environmental effect (95% confidence intervals) from best fitting AE model.

NSSI at age 18 and SA at age 24	
**Univariate estimates**	
NSSI at age 18	
A	0.53 (0.41–0.65)
E	0.47 (0.36–0.59)
SA at age 24	
A	0.30 (0–0.65)
E	0.70 (0.35–1.00)
**Bivariate estimates**	
Phenotypic correlation	0.43 (0.16–0.71)
rA	0.81 (0.27–1.00)
rE	0.19 (0–0.93)
Bivariate A	0.75 (−0.15–1.64)
Bivariate E	0.26 (−0.64–1.15)
**Explained variance in trait 2 by 4 sources**	
From A1	0.20 (0.04–0.35)
From A2	0.10 (0–0.47)
From E1	0.03 (0–0.22)
From E2	0.67 (0.32–0.98)

Confidence intervals are of Wald type, values of the confidence intervals outside the parameter space (i.e., variances <0 and correlations >1) are truncated in the table.

*A* additive genetic effects, *E* non-shared environmental effects, *NSSI* nonsuicidal self-injury, *SA* suicide attempt, *rA* additive genetic correlation, *rE* non-shared environmental correlation.

Additive genetics accounted for 30% of the variance of SA reported at age 24, of which 66.6% were explained by the genetic factors related to NSSI reported at age 18. In other words, a substantial proportion of the genetic risk for SAs at age 24 was explained by genetic factors influencing NSSI reported at age 18.

## Discussion

In this longitudinal study, we examined genetic and environmental influences on the transition between NSSI reported at age 18 and SA reported at age 24 in 3 934 youth, without previous SAs at age 18. NSSI reported at age 18 was highly associated with an increased risk of SA (i.e., a five-fold increase in the odds), for both sexes and independent of other childhood psychopathology. We found a strong genetic correlation between NSSI reported at age 18 and SA reported at age 24. At age 18, the proportion of variance in NSSI explained by genetic factors was 53%, the remaining variance was explained by non-shared environmental factors (47%). At age 24, heritability explained 30% of the variance in suicidal attempt, largely explained by shared genetic factors at age 18.

The finding that NSSI in youth is associated with SA in young adulthood is well-established [2, 28–30]. Our findings also concur with research demonstrating a strong genetic correlation between NSSI and SA in youth [18] and between NSSI and suicidal ideation in adults [19]. Although a strong genetic correlation between two related behaviors is not surprising given the substantial genetic correlation that has been demonstrated between a range of different psychiatric conditions [15–17], our study is the first to our knowledge to show the genetic and environmental influences on the transition of NSSI to SA. The strong genetic contribution to the risk of SA in youth with NSSI suggests that future research should investigate the heritable mechanisms of the association. Examples of such mechanisms are pain threshold and pain regulation. Theories state that repeated NSSI is associated with habituation of inflicting pain to oneself and eventually increased capability for enacting a SA [31]. Individuals with a history of repeated NSSI have indeed been shown to have elevated pain thresholds [32–34] and a more effective central down-regulation of pain [33]. Pain sensitivity and regulation capacity are complex heritable traits that probably have polygenetic origin [35]. A twin study suggests that acquired capability of suicide, referring to fearlessness about death and physical pain, is primarily influenced by genetics but likely results from a combination of both genetic and non-shared environmental factors [36]. Thus, the genetic influence in the transition from NSSI to SA found in the present study might partly be explained by an acquired capability for suicide resulting from an interaction between genetically driven pain tolerance [33] and underlying traits, such as impulsivity and aggression [36], in combination with repeated acts of self-injury which may lead to habituation to pain [32]. Future studies should delineate the genetic and environmental aetiologies involved in pain sensitivity or other potential heritable traits.

From a clinical perspective, the findings stress the importance of identifying vulnerable individuals for evidence-based preventive interventions such as means restriction and safety planning. The results also underscore the significance of monitoring suicidal intent in youth who engage in NSSI. In addition, the presence of NSSI at age 18 is largely affected by non-shared environmental factors. These factors, such as adverse childhood experiences and bullying [37], could be appropriate targets för psychosocial interventions. Addressing these could potentially reduce the risk of future SA. Stressful life events, perceived burdensomeness and belongingness are potential non-shared environmental factors that might contribute to the transition from NSSI to SA [38].

### Strengths and limitations

Our study has several strengths, including but not limited to, a longitudinal design, twins of both sexes, and a nationwide sample. Nevertheless, our results should be interpreted with caution. First, the generalizability from the current twin sample to the general population is not known. Lastly, items measuring NSSI and SA were taken from LHA, asked in the context of aggression. However, the prevalence of NSSI and SA found in the present study are similar to what is generally found in community-based samples [39].

## Conclusions

In conclusion, using a twin sample with repeated measurement, we found evidence that NSSI reported at age 18 was associated with an increased risk of SA between ages 18 and 24, independent of sex and childhood psychopathology. NSSI reported at age 18 had strong genetic correlation with SA reported at age 24.

## Supplementary information

Supplementary Table 1

Supplementary Table 2

## Data Availability

Data may be obtained from a third party and are not publicly available. The Public Access to Information and Secrecy Act in Sweden prohibits individual-level data to be publicly available. Researchers who are interested in replicating this study can apply for individual level data at The Swedish Twin Registry, managed by Karolinska Institutet: https://strdata.se/.

## References

[1] Castellvi P, Lucas-Romero E, Miranda-Mendizabal A, Pares-Badell O, Almenara J, Alonso I, et al. Longitudinal association between self-injurious thoughts and behaviors and suicidal behavior in adolescents and young adults: A systematic review with meta-analysis. J Affect Disord. 2017;215:37–48. 28315579 10.1016/j.jad.2017.03.035

[2] Ohlis A, Bjureberg J, Lichtenstein P, D’Onofrio BM, Fruzzetti AE, Cederlöf M, et al. Comparison of suicide risk and other outcomes among boys and girls who self-harm. Eur Child Adolesc Psychiatry. 2020;29:1741–6. 32056009 10.1007/s00787-020-01490-y PMC7641927

[3] Ribeiro JD, Franklin JC, Fox KR, Bentley KH, Kleiman EM, Chang BP, et al. Self-injurious thoughts and behaviors as risk factors for future suicide ideation, attempts, and death: a meta-analysis of longitudinal studies. Psychological Med. 2016;46:225–36. 10.1017/S0033291715001804 PMC4774896 26370729

[4] Mental health of adolescents. https://www.who.int/news-room/fact-sheets/detail/adolescent-mental-health, 2021, Accessed Date Accessed 2021 Accessed.

[5] Asarnow JR, Porta G, Spirito A, Emslie G, Clarke G, Wagner KD, et al. Suicide attempts and nonsuicidal self-injury in the treatment of resistant depression in adolescents: findings from the TORDIA study. J Am Acad Child Adolesc Psychiatry. 2011;50:772–81. 21784297 10.1016/j.jaac.2011.04.003 PMC3143365

[6] Chesin MS, Galfavy H, Sonmez CC, Wong A, Oquendo MA, Mann JJ, et al. Nonsuicidal self-injury is predictive of suicide attempts among individuals with mood disorders. Suicide Life-Threatening Behav. 2017;47:567–79. 10.1111/sltb.12331 PMC5724372 28211201

[7] Griep SK, MacKinnon DF. Does nonsuicidal self-injury predict later suicidal attempts? a review of studies. Arch Suicide Res. 2022;26:428–46. 32985383 10.1080/13811118.2020.1822244

[8] Guan K, Fox KR, Prinstein MJ. Nonsuicidal self-injury as a time-invariant predictor of adolescent suicide ideation and attempts in a diverse community sample. J Consult Clin Psychol. 2012;80:842–9. 22845782 10.1037/a0029429 PMC3458144

[9] Masi G, Pisano S, Sesso G, Mazzullo C, Berloffa S, Fantozzi P, et al. Persistent non-suicidal self-injury and suicidality in referred adolescents: a longitudinal study exploring the role of cyclothymic temperament. Brain Sci. 2023;13:755. 37239227 10.3390/brainsci13050755 PMC10216602

[10] Whitlock J, Muehlenkamp J, Eckenrode J, Purington A, Baral Abrams G, Barreira P, et al. Nonsuicidal self-injury as a gateway to suicide in young adults. J Adolesc Health. 2013;52:486–92. 23298982 10.1016/j.jadohealth.2012.09.010

[11] Wilkinson P, Kelvin R, Roberts C, Dubicka B, Goodyer I. Clinical and psychosocial predictors of suicide attempts and nonsuicidal self-injury in the Adolescent Depression Antidepressants and Psychotherapy Trial (ADAPT). Am J Psychiatry. 2011;168:495–501. 21285141 10.1176/appi.ajp.2010.10050718

[12] Jacobson CM, Muehlenkamp JJ, Miller AL, Turner JB. Psychiatric impairment among adolescents engaging in different types of deliberate self-harm. J Clin Child Adolesc Psychol. 2008;37:363–75. 18470773 10.1080/15374410801955771

[13] O’Connor RC, Wetherall K, Cleare S, Eschle S, Drummond J, Ferguson E, et al. Suicide attempts and non-suicidal self-harm: national prevalence study of young adults. BJPsych Open. 2018;4:142–8. 29922479 10.1192/bjo.2018.14 PMC6003254

[14] Wilcox HC, Arria AM, Caldeira KM, Vincent KB, Pinchevsky GM, O’Grady KE. Longitudinal predictors of past-year non-suicidal self-injury and motives among college students. Psychological Med. 2012;42:717–26. 10.1017/S0033291711001814 PMC3237933 21906421

[15] Cross-Disorder Group of the Psychiatric Genomics Consortium. Electronic address: plee0@mgh.harvard.edu; Cross-Disorder Group of the Psychiatric Genomics Consortium. Genomic relationships, novel loci, and pleiotropic mechanisms across eight psychiatric disorders. Cell. 2019;179:1469–82.e1411. 31835028 10.1016/j.cell.2019.11.020 PMC7077032

[16] Smoller JW. Disorders and borders: psychiatric genetics and nosology. Am J Med Genet B Neuropsychiatr Genet. 2013;162b:559–78. 24132891 10.1002/ajmg.b.32174

[17] Smoller JW. The genetics of stress-related disorders: PTSD, depression, and anxiety disorders. Neuropsychopharmacology. 2016;41:297–319. 26321314 10.1038/npp.2015.266 PMC4677147

[18] Lim KX, Krebs G, Rimfeld K, Pingault JB, Rijsdijk FV. Investigating the genetic and environmental aetiologies of non-suicidal and suicidal self-harm: a twin study. Psychol Med. 2021;52:1–11. 33558000 10.1017/S0033291721000040 PMC9772908

[19] Maciejewski DF, Creemers HE, Lynskey MT, Madden PA, Heath AC, Statham DJ, et al. Overlapping genetic and environmental influences on nonsuicidal self-injury and suicidal ideation: different outcomes, same etiology? JAMA Psychiatry. 2014;71:699–705. 24760386 10.1001/jamapsychiatry.2014.89 PMC4241464

[20] Sedgwick R, Ougrin D. No better than chance? Developments in predicting adolescent suicide, a commentary on Mars et al. (2018) and Beckman et al. (2018). J Child Psychol Psychiatry. 2019;60:100–1. 30556605 10.1111/jcpp.12982

[21] Anckarsäter H, Lundström S, Kollberg L, Kerekes N, Palm C, Carlström E, et al. The child and adolescent twin study in Sweden (CATSS). Twin Res Hum Genet. 2011;14:495–508. 22506305 10.1375/twin.14.6.495

[22] Larson T, Anckarsäter H, Gillberg C, Ståhlberg O, Carlström E, Kadesjö B, et al. The autism-tics, AD/HD and other comorbidities inventory (A-TAC): further validation of a telephone interview for epidemiological research. BMC Psychiatry. 2010;10:1–11. 20055988 10.1186/1471-244X-10-1 PMC2823676

[23] Mårland C, Lichtenstein P, Degl’Innocenti A, Larson T, Råstam M, Anckarsäter H, et al. The autism–tics, ADHD and other comorbidities inventory (A-TAC): previous and predictive validity. BMC Psychiatry. 2017;17:1–8. 29246205 10.1186/s12888-017-1563-0 PMC5732476

[24] O’Reilly LM, Pettersson E, Quinn PD, Klonsky ED, Lundström S, Larsson H, et al. The association between general childhood psychopathology and adolescent suicide attempt and self-harm: a prospective, population-based twin study. J Abnorm Psychol. 2020;129:364–75. 32271026 10.1037/abn0000512 PMC7179089

[25] Neale MC, Hunter MD, Pritikin JN, Zahery M, Brick TR, Kirkpatrick RM, et al. OpenMx 2.0: Extended structural equation and statistical modeling. Psychometrika. 2016;81:535–49. 25622929 10.1007/s11336-014-9435-8 PMC4516707

[26] Neale M, Cardon LR *Methodology for genetic studies of twins and families*, vol. 67. Springer Science & Business Media 2013.

[27] Kendler KS, Neale MC, Kessler RC, Heath AC, Eaves LJ. A test of the equal-environment assumption in twin studies of psychiatric illness. Behav Genet. 1993;23:21–7. 8476388 10.1007/BF01067551

[28] Bjureberg J, Kuja-Halkola R, Ohlis A, Lichtenstein P, D’Onofrio BM, Hellner C, et al. Adverse clinical outcomes among youths with nonsuicidal self-injury and suicide attempts: a longitudinal cohort study. J Child Psychol Psychiatry. 2022;63:921–28. 10.1111/jcpp.13544 34856636

[29] Bjureberg J, Ohlis A, Ljótsson B, D’Onofrio BM, Hedman-Lagerlöf E, Jokinen J, et al. Adolescent self-harm with and without suicidality: cross-sectional and longitudinal analyses of a Swedish regional register. J Child Psychol Psychiatry. 2019;60:295–304. 30207392 10.1111/jcpp.12967 PMC7379534

[30] Mars B, Heron J, Crane C, Hawton K, Lewis G, Macleod J, et al. Clinical and social outcomes of adolescent self harm: population based birth cohort study. BMJ. 2014;349:g5954. 25335825 10.1136/bmj.g5954 PMC4205277

[31] Hamza CA, Stewart SL, Willoughby T. Examining the link between nonsuicidal self-injury and suicidal behavior: a review of the literature and an integrated model. Clin Psychol Rev. 2012;32:482–95. 22717336 10.1016/j.cpr.2012.05.003

[32] Cummins TM, English O, Minnis H, Stahl D, O’Connor RC, Bannister K, et al. Assessment of somatosensory function and self-harm in adolescents. JAMA Netw Open. 2021;4:e2116853. 34255048 10.1001/jamanetworkopen.2021.16853 PMC8278268

[33] Lalouni M, Fust J, Bjureberg J, Kastrati G, Fondberg R, Fransson P, et al. Augmented pain inhibition and higher integration of pain modulatory brain networks in women with self-injury behavior. Mol Psychiatry. 2022;27:3452–9. 35691963 10.1038/s41380-022-01639-y PMC9708552

[34] Pontén M, Lee M, Khoo S, Nilsson G, Nevin E, Walldén Y, Ougrin D, et al. Pain threshold and pain tolerance in young people with self-injurious behavior: a systematic review and meta-analysis. Psychiatry Res. 2025;351:116638. 40714719 10.1016/j.psychres.2025.116638

[35] Sexton JE, Cox JJ, Zhao J, Wood JN. The genetics of pain: implications for therapeutics. Annu Rev Pharmacol Toxicol. 2018;58:123–42. 28968191 10.1146/annurev-pharmtox-010617-052554

[36] Smith AR, Ribeiro JD, Mikolajewski A, Taylor J, Joiner TE, Iacono WG. An examination of environmental and genetic contributions to the determinants of suicidal behavior among male twins. Psychiatry Res. 2012;197:60–5. 22417928 10.1016/j.psychres.2012.01.010 PMC3376176

[37] Wang YJ, Li X, Ng CH, Xu DW, Hu S, Yuan TF. Risk factors for non-suicidal self-injury (NSSI) in adolescents: A meta-analysis. EClinicalMedicine. 2022;46:101350. 35330803 10.1016/j.eclinm.2022.101350 PMC8938878

[38] Joiner TE. Why people die by suicide. Harvard University Press; 2005.

[39] Gillies D, Christou MA, Dixon AC, Featherston OJ, Rapti I, Garcia-Anguita A, et al. Prevalence and characteristics of self-harm in adolescents: meta-analyses of community-based studies 1990–2015. J Am Acad Child Adolesc Psychiatry. 2018;57:733–41. 30274648 10.1016/j.jaac.2018.06.018

