# Supplementary material for: Genetic and environmental aetiologies of the transition from nonsuicidal self-injury to suicide attempt: a longitudinal twin study
Source: Mol Psychiatry. 2025 Aug 22;30(12):5828–32. doi: 10.1038/s41380-025-03165-z (PMC12602349; doi:10.1038/s41380-025-03165-z)
Supplement: Supplementary file 1 — Supplementary Table 1 [file 41380_2025_3165_MOESM1_ESM.docx]

**Supplementary Table 1**. Observed concordance and discordance in males and females.

|  | NSSI at age 18 | | SA at age 24 | | Tetrachoric correlations | | |
| --- | --- | --- | --- | --- | --- | --- | --- |
|  | Both affected/both unaffected^a^ | Discordant pairs^b^ | Both affected/both unaffected | Discordant pairs | ICC NSSI at age 18 | ICC SA at age 24 | CTCT |
| MZ female | 40/299 | 102 | 2/411 | 28 | 0.61(0.49-0.75) | 0.43 (0.03-0.84) | 0.22(0.03-0.44) |
| MZ male | 11/170 | 40 | 0/209 | 12 | 0.50(0.36-0.66) | 0.39(-0.01-0.81) | 0.19(-0.03-0.40) |
| DZ female | 18/226 | 102 | 0/320 | 26 | 0.24 (0.03-0.46) | -0.46 (-1.04-0.12) | 0.23(-0.01-0.47) |
| DZ male | 6/156 | 43 | 0/194 | 11 | 0.30(-0.03-0.63) | -0.49(-1.46,0.48) | 0.23(-0.01-0.47) |
| DZ female-male | 15/341 | 148 | 2/459 | 43 | -0.01(-0.22-0.21) | 0.15(-0.19-0.50) | -0.54(-0.28-0.17) |

*Note.* NSSI = nonsuicidal self-injury, SA = suicide attempt, ICC = intraclass correlation, CTCT = cross-twin cross-trait, MZ = monocygotic twin, DZ = dizygotic twin.

^a^Number of pairs with both twins affected versus (/) number of pairs with both twins unaffected by the trait.

^b^Number of pairs where one twin was affected with the trait and the other was unaffected.
