# Supplementary material for: Genetic and environmental aetiologies of the transition from nonsuicidal self-injury to suicide attempt: a longitudinal twin study
Source: Mol Psychiatry. 2025 Aug 22;30(12):5828–32. doi: 10.1038/s41380-025-03165-z (PMC12602349; doi:10.1038/s41380-025-03165-z)
Supplement: Supplementary file 2 — Supplementary Table 2 [file 41380_2025_3165_MOESM2_ESM.docx]

**Supplementary Table 2**. Quantitative genetic results from different models.

|  | **SA at age 24** | | |
| --- | --- | --- | --- |
|  | **ACE** | **ADE** | **AE** |
| **AIC** | 3887.788 | 3887.788 | **3881.788** |
| **BIC** | 3869.788 | 3869.788 | 3869.788 |
| **P** | NA | NA | 0.99 |
| **Univariate estimates** | | | |
| NSSI-18 | NSSI at age 18 |  |  |
| A | 0.53 (0.41-0.65) | 0.06 0-0.48) | 0.53 (0.41-0.65) |
| D | NA | 0.51 (0.06-0.94) | NA |
| C | 0.00 | NA | NA |
| E | 0.47 (0.36-0.59) | 0.43 (0.31-0.55) | 0.47 (0.36-0.59) |
|  | SA at age 24 |  |  |
| A | 0.30 (-0.05-0.65) | 0.14 (0-1.16) | 0.30 (0-0.65) |
| D | NA | 0.19 (0-1.38) | NA |
| C | 0.00 | NA | NA |
| E | 0.70 (0.35-1.00) | 0.66 (0.26-1.00 | 0.70 (0.35-1.00) |
| **Bivariate estimates** | | | |
| Phenotypic correlation | 0.43 (0.16-0.71) | 0.43 (0.16-0.71) | 0.43 (0.16-0.71) |
| rA | 0.81 (0.27-1.35) | 1.00 (1.00-1.00) | 0.81 (0.27-1.35) |
| rD | NA | 0.79 (0-2.34) | NA |
| rC | 1.00 | NA | NA |
| rE | 0.19 (0-0.93) | 0.17 (0-1.53) | 0.19 (0-0.93) |
| Bivariate A | 0.75 (-0.15-1.64) | 0.21 (-0.99-1.41) | 0.75 (-0.15-1.64) |
| Bivariate D | NA | 0.59 (-0.76-1.93) | NA |
| Bivariate C | 0.00 | NA | NA |
| Bivariate E | 0.26 (-0.64-1.15) | 0.26 (-0.21-0.62) | 0.26 (-0.64-1.15) |

*Note*. SA = suicide attempt, A = additive genetic effects, C = shared environmental effects, E = non-shared environmental effects, D = non-additive genetic effects, AIC = Akaike information criterion, BIC = Bayesian information criterion, NA = not applicable, NSSI = nonsuicidal self-injury, rA = additive genetic correlation, rD = non-additive genetic correlation, rC = shared environmental correlation, rE = non-shared environmental correlation. Confidence intervals are of Wald type, values of the confidence intervals outside the parameter space (i.e., variances <0 and correlations >1) are truncated in the table.
